# Rare *Shewanella* spp*.* associated with pulmonary and bloodstream infections of cancer patients, China: a case report

**DOI:** 10.1186/s12879-018-3354-8

**Published:** 2018-09-05

**Authors:** Furong Zhang, Yujie Fang, Feng Pang, Shengnan Liang, Xin Lu, Biao Kan, Jianguo Xu, Jinxing Zhao, Yinju Du, Duochun Wang

**Affiliations:** 1 Liaocheng Center for Disease Control and Prevention, Liaocheng, People’s Republic of China; 2 State Key Laboratory of Infectious Disease Prevention and Control, National Institute for Communicable Disease Control and Prevention, Chinese Center for Disease Control and Prevention, Beijing, 102206 People’s Republic of China; 3 Collaborative Innovation Center for Diagnosis and Treatment of Infectious Diseases, Hangzhou, 310003 People’s Republic of China; 4 Center for Human Pathogen Collection, Chinese Center for Disease Control and Prevention, Beijing, 102206 People’s Republic of China; 5 Department of Clinical Laboratory, Qilu Hospital of Shandong University, Jinan, 250012 People’s Republic of China; 6 Department of Clinical Laboratory, Liaocheng People’s Hospital, Liaocheng, 252000 People’s Republic of China

**Keywords:** Rare *Shewanella* spp*.*, Pulmonary and bloodstream infections, Cancer patients, China

## Abstract

**Background:**

Members of *Shewanella* species are opportunistic pathogens that are found in marine environments. Currently more than sixty species have been identified, whereas the most commonly clinical cases associated with *Shewanella* species have involved only two species, i.e., *S. algae* and *S. putrefaciens.* We present two cases of pulmonary and bloodstream infections caused by two rare *Shewanella* spp*.* strains from patients of gastrointestinal cancer.

**Case presentation:**

Two male patients with a history of gastrointestinal cancer presented to hospital with pulmonary and bloodstream infections, respectively. The infective pathogens of both cases were primarily isolated and identified as *Shewanella algae* (case I) and *Shewanella putrefaciens* (case II) by phenotypic features and VITEK 2 system, but they were further confirmed as *Shewanella haliotis* and *Shewanella upenei* by 16S rRNA gene sequence analysis. The major bacterial composition of the bronchoalveolar lavage in case I was also identified as *Shewanella* by 16S rRNA amplicon sequencing analysis. Antimicrobial susceptibility testing showed that the two strains had broad susceptibility, but *S. haliotis* in the case I was resistant to ciprofloxacin and levofloxacin and *S. upenei* in the case II was intermediate to imipenem, piperacillin/tazobactam and ciprofloxacin.

**Conclusions:**

To the best of our knowledge, this is the first cases of the pulmonary and bloodstream infections caused by *Shewanella* spp*.* from clinical patients in mainland China. *Shewanella* as a potential pathogen in China should not be ignored.

**Electronic supplementary material:**

The online version of this article (10.1186/s12879-018-3354-8) contains supplementary material, which is available to authorized users.

## Background

The genus *Shewanella* comprises members of Gram-negative, motile, oxidase-positive, facultative anaerobic and motile rods. Bacteria are widely distributed in nature, mainly in marine environment. Currently the genus composes of more than sixty species (http://www.bacterio.net/shewanella.html); among them, four species have been reported to cause human infections, i.e., *Shewanella putrefaciens, Shewanella algae, Shewanella haliotis* [1] and *Shewanella xiamenensis* [2], whereas the most commonly clinical cases associated with *Shewanella* species have involved only two species, i.e., *S. algae* and *S. putrefaciens* [3]*.* In this study, we identified *S. haliotis* an*d S. upenei* strains isolated from pulmonary tissue infection and bacteremia of clinical patients in Liaocheng, an inland city in China. Those are first reported cases of pulmonary and bloodstream infections caused by *Shewanella* spp. in mainland China. Our study suggests that *Shewanella* spp. is a potential opportunistic pathogen which should not be ignored even outside coastal regions.

## Case presentation

### Case 1

A esophageal cancer patient who was diagnosed as *S. haliotis* pulmonary inflammation.

A 68-year-old male patient was admitted to people’s hospital of Liaocheng city, China, on July 24, 2016, because of “hematemesis for 4 hours”. He had been diagnosed with the operation of esophageal cancer for more than 2 years. His admitted physical examinations were body temperature of 36.3 °C, pulse rate 92 beats/min, breathing of 22 times/min and blood pressure 135/80 mmHg. Nonpalpable enlargement of bilateral neck and supraclavicular lymph nodes, trachea in the middle, pectoral symmetry, visible scars at right chest, clear percussion sound at double lung, auscultation of coarse breath sound, no dry and wet rales, regular rhythm, percussion no pain of the kidney area, negative for shifting dullness and bowel sounds of 3 times/min. His admission diagnosis was esophageal cancer after operation and hypertension. On admission, the auxiliary examinations were performed to determine the source of hematemesis. The painless gastroscopy was carried out, but no obvious abnormalities was observed. The painless bronchoscopic examination revealed posterior basal segment of left lower lobe hemorrhage. Brushing pathology indicated no obvious tumor cells. Thoracic and abdominal enhanced computed tomography scan showed that he had esophageal surgery, bronchitis and emphysema, middle lobe of right lung nodules, right upper lobe and left lower lobe interstitial lesions and the lower lobe of the left lung inflammation. He was given medicine (3 g of cefoperazone/sulbactam was administrated twice a day for 6 days) and therapy of anticancer, anti-inflammatory, rehydration and hemostasis. After six days’ treatment, his symptoms improved and the patient was discharged from the hospital.

**Fig. 1:**
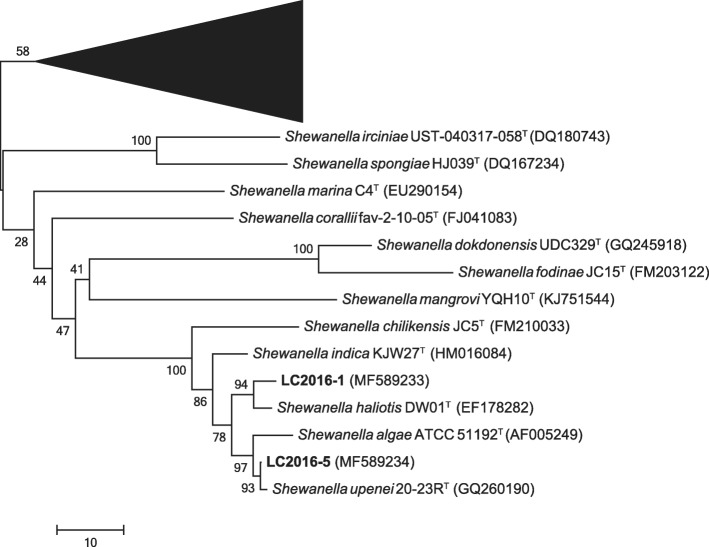
Phylogenetic tree constructed by the neighbour-joining method based on the nucleotide sequences of the 16S rRNA gene. The phylogenetic tree was constructed from an alignment of 1427 nt and all known type strains of the *Shewanella* species were included. Numbers at nodes indicated bootstrap values (percentage of 1000 replicates). Bar, 10 substitutions per nucleotide position. Bold font indicated the strains from case I and II. GenBank accession numbers of 16S rRNA gene sequences were in brackets. The black triangle consisted of fifty-four *Shewanella* species. Species names and GenBank accession numbers of 16S rRNA gene sequences were listed in Additional file 1: Table S1

The bronchoalveolar lavage fluid (BALF) was collected when he received painless bronchoscopic examination and the cell number was over 10^4^ cfu/ml. Sample was streak-inoculated on blood agar medium for bacterial culture. Strains of different phenotypic features at blood plates were isolated and identified as *S. algae, Escherichia coli* and *Klebsiella pneumoniae* by VITEK 2 system using the ID-GN card (boiMérieux). Since only two *Shewanella* species, *S. putrefaciens* and *S. algae,* were registered in the database of VITEK 2 system, the 16S rRNA gene sequence was amplified by a PCR described previously [4]. The PCR product was sequenced and the nucleotide sequence had been deposited at GenBank, under the accession number of MF589233. BLAST analysis of 16S rRNA gene sequence at GenBank showed a similarity of 99.0% with type strain of *S. haliotis* DW01 (accession numbers NR_117770.1). Further phylogenetic analysis with all type sequences of *Shewanella* species available in the GenBank database, confirmed the strain was identified as species of *S. haliotis* (LC2016–1 in Fig. 1)*.*

To further confirm the bacterial community composition and richness of the BALF, sample was subjected to the 16S rRNA amplicon sequencing [5, 6]. The result indicated the bacterial community composition included genera of *Shewanella* (88.34%), *Escherichia* (11.11%) and *Streptococcus* (0.38%), et al., whereas the majority of genus was *Shewanella*.

Antibiotic susceptibility testing was performed by microdilution method on Mueller-Hinton broth. The strain was susceptible to piperacillin/tazobactam (minimal inhibitory concentration, MIC: 8 μg/ml), ceftazidime (1 μg/ml), cefepime (1 μg/ml), amikacin (2 μg/ml), gentamicin (1 μg/ml), imipenem (4 μg/ml), meropenem (4 μg/ml), but was resistant to ciprofloxacin (8 μg/ml) and levofloxacin (8 μg/ml).

### Case 2

A gastric cancer patient who was diagnosed as *S*. *algae* bacteremia.

A 56-year-old man was admitted to people’s hospital of Liaocheng city, China, on 6, Oct. 2016, because of “discomfort of upper abdominal pain for 1 month”. His admitted physical examination included body temperature of 36.1 degrees, pulse rate of 72 beats/min, breathing 18 times/min and blood pressure 140/90 mmHg. Detection of nonpalpable enlargement of bilateral neck and supraclavicular lymph nodes, flat abdomen, no gastrointestinal or peristaltic waves were observed. Soft abdominal muscles, mild tenderness in the upper abdomen and no obvious rebound pain were reported. His liver and spleen did not touch under the rib and no palpable mass was discovered. Negative for shifting dullness, normal bowel sounds and no abnormal of rectal examination were detected. The gastroscope suggested visible ulcer lesions at the cardiac involving gastric fundus and gastric body. The pathological results indicated adenocarcinoma. His admission diagnoses were gastric cancer and hypertension.

On admission, the auxiliary examination were carried out on Oct. 9, 2016. Laparoscopy indicated he was in the late stage tumors without radical resection. He then received intravenous and intraperitoneal chemotherapy, followed by severe bone marrow suppression with blood cells and platelets significantly lower than normal. He was given further treatment of anti infection, nutritional support, rehydration, stimulating granulopoiesis and symptomatic treatment. On Oct. 26, 2016, patients had shortness of breath, heart rate and other symptoms with lung breath sounds rough, and no rales, limbs cold. He was considered the existence of septic shock. He was given non-invasive mechanical ventilation and fluid expansion, colloid, blood transfusion products, anti infection (1 g of imipenem was administrated every 8 h for 7 days), maintain circulation, acid suppression, liver protection, nutritional support, maintenance of water and electrolyte acid-base balance, monitoring blood pressure, heart rate, respiratory function, hour urine volume and bleeding. Patient had severe infection, and the presence of multiple organ dysfunction syndrome (breathing, circulation, gastrointestinal, blood and kidney). Patient and his family members required automatic discharge for hospice care. His discharge diagnoses were multiple organ dysfunction syndrome (respiratory, circulatory, gastrointestinal, blood and kidney), gastric cancer and hypertension.

After appearing septic shock, his blood culture was sampled to separate the bacteria. The microbial growth was detected in both anaerobic and aerobic bottles and the positive reported time were 8.1 and 11.9 h, respectively. Both bottles yielded an uniform Gram-negative bacillus. After 24 h incubation, haemolytic, oxidase-positive yellow colonies grew on blood agar. The strain was identified as *S. putrefaciens* by VITEK 2 system using the ID-GN card (boiMérieux). The 16S rRNA gene sequence of the strain had been deposited at GenBank (accession number: MF589234). BLAST analysis at GenBank showed a similarity of 100.0% with *S. upenei* strain VITVAGJ (accession numbers KP090164.1). Further phylogenetic analysis with all type sequences of *Shewanella* species available in the GenBank database, confirmed the strain belonged to species of *S. upenei* (LC2016–5 in Fig. 1)*.* On the day of blood sampling, his peritoneal drainage fluid was also collected and cultured using the same identification methods, and the results of bacterial identification and drug sensitivity were consistent with that of blood.

Antibiotic susceptibility testing was performed by microdilution method on Mueller-Hinton broth. The strain was susceptible to aztreonam (1 μg/ml), ceftazidime (1 μg/ml), cefepime (1 μg/ml), amikacin (2 μg/ml), gentamicin (1 μg/ml) and levofloxacin (1 μg/ml), but was intermediate to imipenem (8 μg/ml), piperacillin/tazobactam (64 μg/ml) and ciprofloxacin (2 μg/ml).

## Discussion and conclusions

In this study, we reported the pulmonary and bloodstream infections caused by two rare *Shewanella* spp. from clinical patients in mainland China. In humans, the majority of *Shewanella*-associated syndromes involves the skin and soft-tissue infections [7–9], followed by bloodborne illnesses [10] and infections of the biliary tree [11, 12]. In recent years, *Shewanella* has also been reported to be associated with food poisoning [13]. Diseases associated with the respiratory tract are rare and *Shewanella* strains have been recovered from sputum of a patient with chronic obstructive airway disease [14], a lung aspirate of a patient with lung tumor [15] and BALF specimens of two patients with ventilator-associated pneumonia [16, 17]. In additional, *Shewanella* have also been thought to play important roles in other cases of empyema or pneumonia [14, 18]. However, among all above cases, multi-pathogens were isolated from the same samples, making the exact role of each organism played in the pathogenicity unclear. In this study of case 1, by 16S rRNA amplicon sequencing, we identified the majority of bacterial community composition of the BALF was *Shewanella,* which strongly suggested that *Shewanella* as the primary pathogen might play a significant contributory role in the pulmonary infection.

Four *Shewanella* species have been confirmed the relevance to clinical infections, i.e., *S. algae*, *S. putrefaciens*, *S. haliotis* [1] and *S. xiamenensis* [2]. Species of *S. haliotis* was first isolated from the gut microflora of abalone in 2007 [19]. Patients associated with *S. haliotis* infection cases have been reported in Japan [20], Thailand [1] and Spain [21]. Cases are involved in phlegmonous inflammation as well as severe soft tissue infections from legs and cellulitis. In this study, the etiology of infection in the case 1 was identified as *S. haliotis* and our report is the first pulmonary infection associated with *S. haliotis* from patient of gastrointestinal cancer from inland city, China. Meanwhile, bloodstream infection associated with *Shewanella* strains have involved only two species, i.e., *S. algae* [10, 22, 23] and *S. putrefaciens* [8, 24]. Species of *S. upenei* was isolated from intestine of bensasi goatfish in 2011 [25], and there have been no report of clinical infection related to *S. upenei.* Our study of case 2 is the first reported of bloodstream infection associated with this rare *Shewanella* species, which was isolated from clinical patient.

Geographically, countries and regions that have reported clinical infections of *Shewanella* include Southeast Asia, Southern Europe, and South Africa. In Asia, *Shewanella*-associated infections have been reported in Thailand [1], Japan [26] and Taiwan [11] of China. Risk factors of human infections attributed to the genus *Shewanella* occur in warmer climates. The majority of cases occurs in coastal areas and most are related to exposure to *Shewanella* species present in seawater and seafood [27]. *Shewanella* illnesses are also typically found in middle-aged to older persons [12]. In this study, the two cases occurred in July and October at Liaocheng, an inland city in China and our retrospective survey reveals the two patients did not establish contact with coastal areas or seafood, although the certain source of infections was indeterminate. The investigation suggests that in addition to coastal areas, *Shewanella* as a potential pathogen at inland areas should not be ignored.

*Shewanella* species in the two cases were primarily identified as *S. algae* and *S. putrefaciens* by VITEK 2 system using the ID-GN card (boiMérieux), and then were confirmed as *S. haliotis* and *S. upenei* by 16S rRNA sequence analysis. This misidentification has also been reported in other studies. A retrospective investigation of *Shewanella* bacteremia in patients with hepatobiliary disease from Taiwan, China indicated that, five out of nine *Shewanella* strains were confirmed as the *S. haliotis* using 16S rRNA sequencing analysis [11]. Byun JH et al. [26] reported 19 strains which were identified as *S. algae* by using VITEK 2 and Matrix-assisted laser desorption/ionization time-of-flight (MALDI-TOF) mass spectrometry, whereas 16S rRNA analysis identified 10 isolates as *S. algae* and 9 isolates as *S. haliotis*. The reason is that only two *Shewanella* species, *S. putrefaciens* and *S. algae* were registered in the database of VITEK 2 system and MALDI-TOF mass spectrometry. Therefore, to identify *Shewanella* at species level, it should also include the 16S rRNA gene sequence analysis. With the surveillance of *Shewanella* species*,* we speculate that more *Shewanella* species in related to the clinical infections will be reported except for the species cited above such as *S. upenei* reported in this study. It is not yet known whether the pathogenicity and severity of the infections are inconsistent with different species of *Shewanella*, therefore, it is an urgent call for surveillance and control activities against *Shewanella* infections*.*

## Additional file

Additional file 1: **Table S1.** *Shewanella* species and GenBank accession numbers of 16S rRNA gene sequences of the black triangle in Fig. 1. (DOC 62 kb)

## Abbreviations

BALF: Bronchoalveolar lavage fluid
MALDI-TOF: Matrix-assisted laser desorption/ionization time-of-flight
MIC: Minimal inhibitory concentration
