# Supplementary material for: Rare Shewanella spp. associated with pulmonary and bloodstream infections of cancer patients, China: a case report
Source: BMC Infect Dis. 2018 Sep 5;18:454. doi: 10.1186/s12879-018-3354-8 (PMC6125870; doi:10.1186/s12879-018-3354-8)
Supplement: Supplementary file 1 — Table S1. Shewanella species and GenBank accession numbers of 16S rRNA gene sequences of the black triangle in Fig. 1. (DOC 62 kb) [file 12879_2018_3354_MOESM1_ESM.doc]

**[*BMC Infectious Diseases*]**

**Supplementary data**

**Rare *Shewanella* spp*.* associated with pulmonary and bloodstream infections of cancer patients, China: a case report**

Furong Zhang^1†^, Yujie Fang^2,3,4†^, Feng Pang^5,6†^, Shengnan Liang^1^, Xin Lu^2,3^, Biao Kan^2,3^, Jianguo Xu^2,3^, Jinxing Zhao^1^, Yinju Du^1^*, Duochun Wang^2,3,4^*

^1^Liaocheng Center for Disease Control and Prevention, Liaocheng, P.R. China.

^2^State Key Laboratory of Infectious Disease Prevention and Control, National Institute for Communicable Disease Control and Prevention, Chinese Center for Disease Control and Prevention, Beijing, P.R. China.

^3^Collaborative Innovation Center for Diagnosis and Treatment of Infectious Diseases, Hangzhou, P.R. China.

^4^Center for Human Pathogen Collection, Chinese Center for Disease Control and Prevention, Beijing, China.

^5^Department of Clinical Laboratory, Qilu Hospital of Shandong University, Jinan, P.R. China

^6^Department of Clinical Laboratory, Liaocheng People's Hospital, Liaocheng, P.R. China

Running title: Rare *Shewanella* spp.; Pulmonary and Bloodstream Infections; China.

Keywords: *Shewanella* spp*.*; Pulmonary and bloodstream infections; China.

^†^ Equal contributors

*Correspondence Author: Duochun Wang, wangduochun@icdc.cn; Yingju Du, lccdcdyj@126.com.

**Table S1**. *Shewanella* species and GenBank accession numbers of 16S rRNA gene sequences of the black triangle in figure 1.

| Species | Acession no |
| --- | --- |
| *Shewanella marinintestina* IK-1^T^ | AB081757 |
| *Shewanella sairae* SM2-1^T^ | AB081762 |
| *Shewanella schlegeliana* HRKA1^T^ | AB081760 |
| *Shewanella pneumatophori* SCRC-2738^T^ | AB204519 |
| *Shewanella halifaxensis* HAW-EB4^T^ | AY579751 |
| *Shewanella pealeana* ANG-SQ1^T^ | AF011335 |
| *Shewanella abyssi* c941^T^ | AB201475 |
| *Shewanella gelidimarina* ACAM 456^T^ | U85907 |
| *Shewanella fidelis* KMM 3582^T^ | AF420312 |
| *Shewanella kaireitica* c931^T^ | AB094598 |
| *Shewanella psychrophila* WP2^T^ | AJ551089 |
| *Shewanella surugensis* c959^T^ | AB094597 |
| *Shewanella piezotolerans* WP3^T^ | AJ551090 |
| *Shewanella benthica* ATCC 43992^T^ | X82131 |
| *Shewanella violacea* DSS12^T^ | D21225 |
| *Shewanella hanedai* ATCC 33224^T^ | U91590 |
| *Shewanella woodyi* MS32^T^ | U91590 |
| *Shewanella atlantica* HAW-EB5^T^ | AY579752 |
| *Shewanella canadensis* HAW-EB2^T^ | AY579749 |
| *Shewanella sediminis* HAW-EB3^T^ | AY579750 |
| *Shewanella waksmanii* KMM 3823^T^ | AY170366 |
| *Shewanella aquimarina* SW-120^T^ | AY485225 |
| *Shewanella loihica* PV-4^T^ | DQ286387 |
| *Shewanella marisflavi* SW-117^T^ | AY485224 |
| *Shewanella algidipiscicola* S13^T^ | AB205570 |
| *Shewanella colwelliana* ATCC 39565^T^ | AY653177 |
| *Shewanella gelidii* RZB5-4^T^ | KR080702 |
| *Shewanella electrodiphila* MAR441^T^ | FR744784 |
| *Shewanella olleyana* ACEM 9^T^ | AF295592 |
| *Shewanella japonica* KMM 3299^T^ | AF145921 |
| *Shewanella pacifica* KMM 3597^T^ | AF500075 |
| *Shewanella donghaensis* LT17^T^ | AY326275 |
| *Shewanella livingstonensis* NF22^T^ | AJ300834 |
| *Shewanella vesiculosa* M7^T^ | AM980877 |
| *Shewanella arctica* IR12^T^ | GU564402 |
| *Shewanella frigidimarina* ACAM 591^T^ | U85903 |
| *Shewanella aestuarii* SC18^T^ | JF751044 |
| *Shewanella denitrificans* OS217^T^ | AJ311964 |
| *Shewanella algicola* ST-6^T^ | FJ903681 |
| *Shewanella gaetbuli* TF-27^T^ | AY190533 |
| *Shewanella basaltis* J83^T^ | EU143361 |
| *Shewanella inventionis* KX27^T^ | KT781407 |
| *Shewanella glacialipiscicola* T147^T^ | AB205571 |
| *Shewanella morhuae* U1417^T^ | AB205576 |
| *Shewanella baltica* NCTC 10735^T^ | AJ000214 |
| *Shewanella oneidensis* MR-1^T^ | AF005251 |
| *Shewanella xiamenensis* S4^T^ | FJ589031 |
| *Shewanella decolorationis* S12^T^ | AJ609571 |
| *Shewanella seohaensis* S7-3^T^ | GU944672 |
| *Shewanella hafniensis* P010^T^ | AB205566 |
| *Shewanella profunda* LT13a^T^ | AY445591 |
| *Shewanella putrefaciens* ATCC 8071^T^ | X82133 |
| *Shewanella amazonensis* SB2B^T^ | AF005248 |
| *Shewanella litorisediminis* SMK1-12^T^ | JQ824139 |
